# An Investigation into the Pathomechanism and Prognostic Markers for Predicting the Outcome of Patients with Moderate and Severe Aortic Stenosis with or Without Intervention

**DOI:** 10.3390/jcm14020446

**Published:** 2025-01-12

**Authors:** Attila Kardos, Tarun Mittal

**Affiliations:** 1 Translational Cardiovascular Research Group, Department of Cardiology, Milton Keynes University Hospital NHS Trust, Milton Keynes MK6 5LD, UK; 2 Heart Division, Royal Brompton and Harefield Hospitals, Guy’s and St Thomas’ NHS Foundation Trust, London SE1 7EH, UK; t.mittal@rbht.nhs.uk; 3 Faculty of Medicine, National Heart & Lung Institute, Imperial College London, London SW3 6LY, UK

Our understanding of the impact of aortic valve stenosis (AS) on patient outcomes irrespective of its severity has changed since the advent of populational databases on AS following the landmark publication by Ross and Braunwald in 1968 [1]. The Australian NASH echo database has shown the prognostic significance of AS from sclerotic aortic valve disease to different grades of AS, highlighting the correlation of worse outcomes with the severity of AS [2,3]. The pathophysiology of AS shares similarities to that of atherosclerosis, and its early detection, personalized prognostication and appropriate intervention constitute an exciting field of research. According to available data, once AS has reached a severe grade in patients with or without symptoms, open-heart or keyhole surgical techniques are the available options to reverse prognosis [4,5,6]. However, data on the best management strategy for non-severe AS are scarce [7]. The timing of intervention and association with survival is a matter of active debate with recent RCT showing no conclusive benefit (e.g., TAVR-Unload, [8]). A new area of interest among calcific aortic valve stenosis/disease is downing. Treating the disease with effective means is essential, but understanding how the disease develops and addressing those mechanisms to mitigate development or reduce progression are now the forefront of researchers’ interest. The complex nature of disease development, from mechanical forces, inflammation and platelet activation to fibrocalcific changes and cell signaling pathways, is a crucial aspect to understand as at the right level, we can avoid disease development. Therapeutic prevention should be a holistic approach, and the ultimate valve intervention should only be the last resort. This Special Issue on AS invites researchers to evaluate the pathomechanism of this disease and identify prognostic markers to predict patient outcomes prior to the development of severe AS and after valve intervention. This Special Issue aims to make a small contribution to the vast knowledge in this scientific field.

The influence of AS and wall shear stress on platelet function has emerged as an important area of research. Banka and collaborators eloquently summarize in their review the role of platelets in the development of AS (Contribution 1). Platelet activation and aggregation, caused by altered hemodynamic in AS, can contribute to inflammation, thrombus formation and disease progression, which in turn result in changes in wall shear stress. Turbulent blood flow and shear stress activate the endothelium, which in turn promotes the adhesion and activation of platelets. Activated platelets release cytokines and chemokines that promote the development of inflammation within the aortic valve. Platelets can liberate mediators, e.g., bioactive lipids such as LPA, that in turn can enhance calcification. Activated platelets promote an osteogenic pathomechanism and the progression of AS through activation of the P2RY1—GPIIb/IIIa-LPA pathway. Antiplatelet therapy aims to prevent platelet activation and aggregation, reducing the risk of thrombotic events. In the context of aortic stenosis, the benefits of antiplatelet therapy have been investigated, although evidence of its clinical benefit is still lacking. Given the heterogeneity of AS patients, an individualized approach to antiplatelet therapy is essential. Patient factors, such as age, comorbidities, bleeding risk and concomitant medications, should be carefully considered when a recommendation for antiplatelet treatment is made. Further research and clinical trials are necessary to understand the underlying molecular mechanisms linking altered wall shear stress to platelet dysfunction, as well as to develop targeted therapies and optimize management approaches in AS.

Sex differences in terms of symptoms, disease severity and the chosen intervention of AS are a major concern. This topic was investigated by Sevilla and colleagues in an attempt to identify differences in sex that predict long-term outcomes (Contribution 2). Interestingly, they found no difference in all-cause death between sexes, but after a successful aortic valve intervention, women had more frequent hospital admissions due to progressive heart failure compared to men. The independent variables predicting heart failure hospitalization in this cohort were age, previous AF or heart failure, and pre-intervention left ventricular end-diastolic diameter, in addition to sex. Their results may encourage further research on the link between sex differences and patient outcomes.

A German research group has investigated whether patients with moderate AS have a similar pattern of a reduced global myocardial work index (GWI), defined as value below 1951 mmHg%, and if so whether it can predict the prognosis, as in those with severe AS (Contribution 3). They recruited 103 moderate-AS patients with no cardiovascular symptoms and followed up with a transthoracic echocardiogram every 6 months. By the end of the 3-year follow-up, 37 patients underwent aortic valve intervention, whereas 66 remained in the non-operated cohort. The time to valve replacement analysis showed no difference between the groups over the 3 year follow-up. Interestingly, none of the echocardiographic parameters differentiated between patients, except for the mean transvalvular gradient and the LV mass, both of which showed higher values in the valve intervention group. They concluded that the GWI is not a reliable prognostic parameter for predicting AVR necessity in patients with moderate AS. This small study is provides further evidence that can aid in finding imaging biomarkers that can improve risk stratification and recognize predictors of outcomes in patients with moderate AS.

An interesting research question was investigated in Reichl et al.’s study regarding whether the improvement in LV systolic function (by LV ejection fraction) after TAVR has prognostic value (Contribution 4). They recruited 385 severe-AS patients with moderately–severely reduced LVEF. They found that TAVR led to long-term improvement in LVEF. Patients who showed early improvement in LVEF after TAVR had lower mortality rates after 1 year and a reduction in mortality and cardiovascular events at 5 years. Interestingly, the multivariate logistic regression analysis did not identify any co-variable that was associated with the prediction of LVEF improvement. Further research is essential to elucidate the underlying mechanisms leading to sustained LVEF improvement in this cohort.

Finally, in their retrospective study, Petrovic and coworkers aimed to evaluate the impact of AF on mortality in 1070 patients with moderate and severe AS over a follow-up period of approximately 5 years, irrespective of the treatment method used (Contribution 5). Their study confirmed that AF predicts all-cause mortality in both moderate- and severe-AS patients. Patients with severe AS and AF had the worst outcome compared to those with severe AS but with a normal sinus rhythm (SR). The presence of AF determined a worse outcome irrespective of the degree of AS. AF and SR patients were different in terms of several clinical and echocardiographic variables, which is characteristic of the general AF population with no VHD. Their study also highlights an interesting observation that can generate several further research questions.

We believe this Special issue has highlighted still unknown evidence related to risk stratification for patients with moderate and/or severe AS who are considered for valve intervention and can guide more precise selection of these patients. The pathophysiological consequence of AV stenosis-related local hemodynamic effects on platelet activation gives an insight into the pathomechanism. We are grateful to the authors who contributed to this interesting Special Issue on aortic valve stenosis.
